# Surgery of the Gummatous Growths of the Nasal Cavities

**Published:** 1889-01

**Authors:** George B. Hope

**Affiliations:** New York


### THE SURGERY OF GUMMATOUS GROWTHS OF THE NASAL CAVITIES.

BY GEORGE B. HOPE, M. D., OF NEW YORK.

On the subject of "The surgery of gummatous growths of the nasal cavities," an article appearing in the November copy of the Record, I take the liberty of offering a few words, mainly in opposition to the conclusion reached with regard to the rational treatment advocated.

Syphilitic gummata of the septum, or arising from the bodies of the turbinated, as is truly stated, are among the rarest of the sequellae of syphilis. Ulcerations of the so-called "tertiary" period, with their characteristic sloughy and indurated sores are not of infrequent occurrence, but well defined gummata seem to show a decided preference to tissues of large expanse or less active vascular supply. During the past year, among over a thousand cases in a hospital service devoted solely to disease of the throat and nose, there has appeared but one instance of the disease in point. Not that this is of necessity a legitimate reason against accepting the four cases cited in the article, other than a repeated witness in favor of the very exceptional type of this disease. In consequence, the fact of syphilis being concurrent should hardly be confirmatory in more than a passing degree, unless the conditions otherwise were so marked as to be conclusive, nor should the feature of recent origin of the obstructive formation add greatly to the argument, inasmuch as glandular and sympathetic enlargements are particularly prone to manifest themselves under these circumstances. In following the subject a step farther, the radical nature of the operation in producing a considerable laceration of tissue without encouraging an extension of the morbid action seems to me to point yet more unmistakably against the theory that would justify a well-founded diagnois of gummatous infection.

Syphilis, particularly in the stage of neoplastic formations, is rightfully termed "specific,"—in the sense that treatment under the iodides reaches a resolution that is practically universal; while many among us have been taught by practical experience that incising a tumor of this kind converts it into a slow and suppurating wound with but little prospect of cicatrization until at last all the elements of its formations have been discharged and the extension of the area involved greatly enlarged. In this process, then, we should be ready to witness a spreading ulceration by so much the more likely to lead to the destruction of bone substance and important parts of the nasal passage, without a corresponding benefit, either in time gained or comfort secured. Examples of phagedenic ulcers running an almost uncontrolled course are not unknown as the consequence of injudicious interference, where even a naturally healthy process of repair in wounds of these parts yields so slowly to treatment, owing to the mucous discharge whic hfouls and irritates the surfaces exposed to its contact.

Is it not wise when the temptation comes to operate on this rare class of cases to consider the distinction between a tissue of permanent, malignant or infectious character, and one where the intrinsic disposition under appropriate medicinal treatment would lead to resolution, and with this in view, to choose, even though the less immediate and the less brilliant, at least, the safer course to the *patient and the surer to the practitioner?*

---

The author of the paper above referred to replies:

I was well aware that I was treading new ground when I first operated on a gummatous tumor for the purpose of relieving nasal stenosis and I did it with fear and trembling; but the ulceration that I expected did not follow, and I operated the next three times with a little less but still much fear. The expected ulceration followed the operation in but one of these cases, and, while it was hard to control, it did not prove insurmountable. I agree with Dr. Hope fully in all his strictures upon hasty operations for nasal gummatous growths. I did not, in the article referred to, recommend that this operation should be resorted to hastily, indeed were another case placed in my hands I should even now hesitate to make the operation. My object in reading the paper before the American Rhinological Association was to recount my rather unique experience in meeting with these four cases in so few years, quite as much as to bring out the fact that instruments can be used (at least in some cases, I do not advise it in all) without the direful sequences that have heretofore been feared. I also concur fully with the doctor on the rarity of gummatous nasal growths. His one case in a thousand, was not proportionally so small as my four cases, occurring in the five to six thousand cases of nasal and throat diseases that I have seen during the last nine years.

If the doctor's criticism of my paper will have effected its purpose in making all who contemplate the operation hesitate, then I am glad he has written it, since I would advise it only where the symptoms are urgent, and all other means have been tried.

A. G. HOBBS, M. D.,
Atlanta, Ga.

